# FOXD3 is a tumor suppressor of colon cancer by inhibiting EGFR-Ras-Raf-MEK-ERK signal pathway

**DOI:** 10.18632/oncotarget.13790

**Published:** 2016-12-03

**Authors:** Kun Li, Qunfeng Guo, Jun Yang, Hui Chen, Kewen Hu, Juan Zhao, Shunxin Zheng, Xiufeng Pang, Sufang Zhou, Yongyan Dang, Lei Li

**Affiliations:** ^1^ Department of Biochemistry and Molecular Biology, Guangxi Medical University, Nanning, Guangxi, China; ^2^ National Center for International Research of Biological Targeting Diagnosis and Therapy, Guangxi Key Laboratory of Biological Targeting Diagnosis and Therapy Research, Guangxi Medical University, Nanning, Guangxi, China; ^3^ Shanghai Key Laboratory of Regulatory Biology, Institute of Biomedical Sciences, School of Life Sciences, East China Normal University, 500 Dongchuan Road, Shanghai, China; ^4^ Department of Orthopedics, Changzheng Hospital, The Second Military Medical University, Shanghai, People's Republic of China

**Keywords:** FOXD3, colon cancer, EGFR, Ras/Raf/MEK/ERK signal pathway

## Abstract

Forkhead box D3 (FOXD3), as a transcriptional repressor, is well known to be involved in the regulation of development. Although FoxD3 is associated with several cancers, its role in colon cancer and the underlying mechanism are still unclear. Here, we first showed that *FOXD3* knockdown dramatically increased the proliferation of human colon cancer cells, enhanced cell invasive ability and inhibited cell apoptosis. *In vivo* xenograft studies confirmed that the *FOXD3*-knockdown cells were more tumorigenic than the controls. Silencing FOXD3 markedly activated EGFR/Ras/Raf/MEK/ERK pathway in human colon cancer cells. In addition, blocking EGFR effectively decreased the activity of MAPK induced by *FOXD3* knockdown. In human cancer tissue, the expression of FOXD3 was reduced, however, the EGFR/Ras/Raf/MEK/ERK pathway was activated. Our study indicates that FOXD3 may play a protective role in human colon formation by regulating EGFR/Ras/Raf/MEK/ERK signal pathway. It is proposed that FOXD3 may have potential as a new therapeutic target in human colon cancer treatment.

## INTRODUCTION

Colon cancer, also known as colorectal cancer, is the most common type of gastrointestinal cancer. Most colon cancers develop first as polyps, which are abnormal growths inside the colon or rectum that may become cancerous later. Colon cancer usually spreads not only to the liver but also to the lungs. A progression from adenoma to carcinoma was supported by the demonstration of mutations in genes of *APC*, *K-RAS*, *P53* and *DCC*. All the mutations are thought to be of importance, but are not able successfully to account for all CRCs [1]. In the instance of mutated APC, the β-catenin pathway remains constitutively active leading to Tcf/Lef associated gene transcription such as *c-myc* and *cyclinD1*, which play an important role in neoplastic transformation [2]. Peroxisome proliferator-activated receptor gamma (PPARγ) is another tumor suppressor in colorectal carcinogenesis [3]. Mice with a heterozygous deletion of *PPARγ* (*PPARγ*^+/−^) have an increased tendency to develop carcinogen-induced colon cancer [4]. Therefore, colorectal carcinogenesis is a complex multistep process involving abnormal epithelial cell proliferation.

Forkhead box D3, also known as FOXD3, is a forkhead protein encoded the *FOXD3* gene, which functions as a transcriptional repressor [5]. It is well known to be involved in the regulation of development. A study showed that FOXD3 is required for maintenance of multipotent embryonic cells of the early mouse embryo [6]. Also, FOXD3 is reported to be an essential Nodal-dependent regulator of zebrafish dorsal mesoderm development [7]. FOXD3 could selectively specify premigratory neural crest cells for a neuronal, glial or cartilage fate and regulate neural crest cell migration [8]. Recently, FOXD3 was found to be associated with cancer development. Silencing *FOXD3* in lung cancer cell lines stimulated cell growth and inhibited cell apoptosis [9]. In melanoma, FOXD3 increases PAX3 expression and contributes to melanoma progression [10]. FOXD3 expression leads to a significant decrease in melanoma cell migration that can be efficiently reversed by the overexpression of TWIST1 [11]. FOXD3 is also a novel tumor suppressor that affects growth, invasion, metastasis and angiogenesis of neuroblastoma [12]. In hepatocellular carcinoma, FOXD3 directly binds to the promoter of miR-137 and activates its transcription, while miR-137 exerted its anti-tumor activity via inhibiting the AKT2/mTOR pathway [13]. However, little information is available concerning the role of FOXD3 in influencing colon cancer formation.

The aim of the study was to investigate whether *FOXD3* deficiency can influence the growth and apoptosis of human colon cancer cells. In addition, the molecular mechanism by which FOXD3 play its role in colon cancer was studied *in vitro and in vivo*.

## RESULTS

### Silencing *FOXD3* increased colon cancer cell proliferation

**Figure 1:**
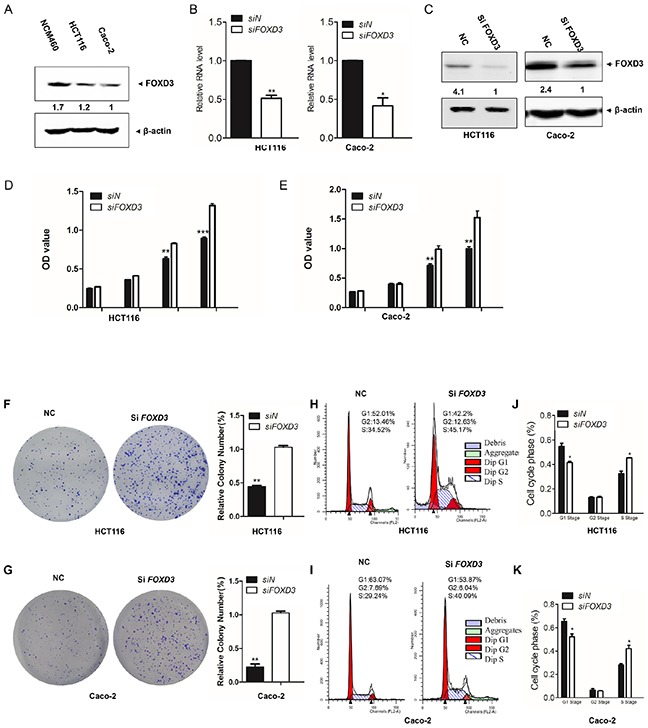
FOXD3 knockdown increased human colon cancer cell proliferation in human colon cancer cells **A**. Western blotanalysis of FOXD3 protein blanket expressionin in NCM460, HCT116 and Caco-2 cells. **B**. HCT116 and Caco-2 cells were transfected with blanket siRNA against *FOXD3* or the negative control (non-targeting) siRNA for 72 hours. *FOXD3* mRNA was measured by blanket Real time PCR. n=3, *p < 0.05. **C**. Western blot blanket analysis of FOXD3 protein blanket expression in HCT116 and Caco2 cells treated with scrambled siRNA (control) or *FOXD3* siRNA. **D** and **E**. After 72 hours post transfection with siRNA against *FOXD3* or the negative control (non-targeting) siRNA, HCT116 and Caco-2 cell viability was monitored by MTT assay at the indicated times. **F** and **G**. Soft-agar colony formation and statistical analysis for HCT116 cells (C) and Caco-2 cells (D) transfected with scrambled siRNA (negative control, NC) and *FOXD3-*siRNA, respectively. The experiments were repeated independently for three times. Results represent mean ± SD (n = 6).*p<0.05, **p<0.01, ***p<0.001, siControl versus si*FOXD3*.

In order to determine the function of FOXD3 in colon cancer, we compared FOXD3 expression levels between colon cancer cells and normal colon cells. The results showed that the expression of FOXD3 was markedly increased in NCM460 cells than that in HCT116 and Caco-2 colon cancer cells (Figure 1A). Next, we knocked down *FOXD3* expression in HCT116 and Caco-2 human colon adenocarcinoma cell lines. Realtime PCR and Western blot showed that *FOXD3* mRNA and protein levels were markedly reduced after transfection of siRNA (Figure 1B and 1C). The MTT experiment showed that cell growth was markedly increased in both HCT116 and Caco-2 cells after *FOXD3* knockdown (Figure 1D and 1E). We further used soft agar colony formation assay to compare cell grow of HCT116 and Caco-2 cells with or without *FOXD3* siRNA (Figure 1F and 1G). Consistent with MTT results, knockdown of *FOXD3* significantly increased cell proliferation compared with the controls, indicating a tumor suppressor potential for FOXD3.

For cell cycle analysis, the percentages of cells in G1-phase were reduced in FOXD3 knockdown cells (Figure 1H, 1I). However, knockdown of FOXD3 induced the accumulation of cells in the S phase in HCT116 and Caco-2 cells (Figure 1J, 1K).

### Silencing *FOXD3* decreased cell apoptosis

**Figure 2:**
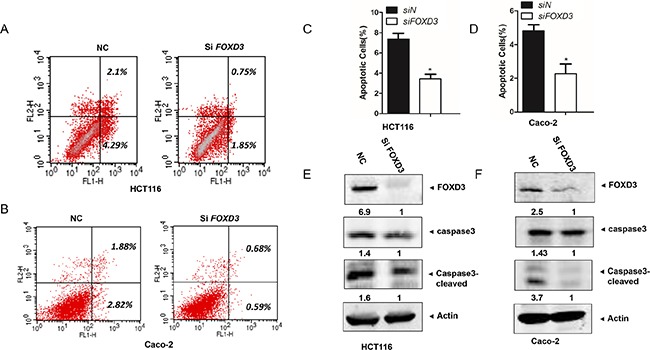
Knockdown FOXD3 decreased human colon cancer cell apoptosis **A** and **B**. HCT116 and Caco-2 cells were collected and analyzed for annexin-V and PI labelling. The percentage of cell that were labelled with annexin-V (primary apoptosis) and with annexin-V plus PI (secondary apoptosis) was measured. The fraction of the total percentages that showed only annexin-V labeling and that showed annexin-V plus PI labelling are shown. **C** and **D**. The ratios of apoptotic cells to all cells were quantified on the basis of three independent experiments. Data are expressed as mean ± SD (n = 3).*p<0.05, siControl versus si*FOXD3*. **E** and **F**. Cells were treated with scrambled siRNA (control) or *FOXD3* siRNA and then collected for Western Blot analysis. Cleavages of caspase-3 were induced in HCT116 cells (C) and Caco-2 cells (D) by *FOXD3* knockdown. β-Actin was used as the internal control.

Analysis of apoptotic cells by flow cytometry revealed an obvious decrease of cell apoptosis in HCT116 and Caco-2 cells after knockdown of *FOXD3*. In HCT116 cell line, the percentages of apoptotic cells (Annex V^+^ PI^+^) undergoing late apoptosis were 0.75% in sh*FOXD3* cells, lower than the negative control cells (2.1%). The population of cell (Annex V^+^ PI^−^) undergoing early apoptosis was also remarkably reduced in sh*FOXD3* cells (1.85%) compared with the controls (4.29%) (Figure 2A). Caco-2 cells with *FOXD3* knockdown also exhibited lower level of cell apoptosis (0.68% for AV^+^ PI^+^ and 0.59% for AV^+^ PI^−^) than their respective control groups (1.88% and 2.82%) (Figure 2B). Thus, knockdown of *FOXD3* significantly decreased the apoptosis of colon cancer cells compared with the controls (Figure 2C, 2D). Since caspase activation is one of the characteristic symbols of cell apoptotic process, we also detected caspase3 activity in human colon cancer cells. As expected, the cleaved caspase 3 was markedly decreased in HCT116 and Caco-2 cells with *FOXD3* silencing compared with their control groups (Figure 2E and 2F). The results suggested that FOXD3 acted in inducing cell apoptosis.

### Knockdown of *FOXD3* increased cell invasive ability and mesenchymal attributes

**Figure 3:**
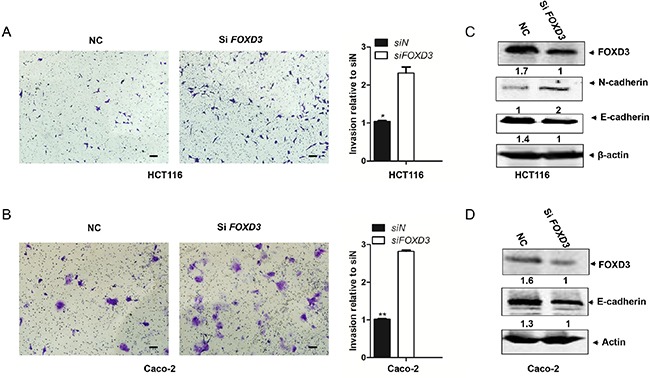
Knockdown FOXD3 increased human colon cancer cell metastasis **A** and **B**. HCT116 and Caco-2 cells were transduced with *FOXD3* siRNA or siControl for 48h and the transwell invasion experiments and quantitative assay were performed. The cells were allowed to migrate for 24 h at 37°C. Data are expressed as mean ± SD (n = 3). (Scale bar, 100μm; original magnification, ×10).*p<0.05, **p<0.01, siControl versus si*FOXD3*. **C** and **D**. Cells were treated with scrambled siRNA (control) or *FOXD3* siRNA and then collected for Western Blot analysis. Cells were lysed 72 h post-infection with shRNAs and analyzed by immunoblotting with the antibodies against N-cadherin, E-cadherin and FOXD3.

As knockdown of *FOXD3* promoted cell proliferation and inhibited cell apoptosis, we further detected whether FOXD3 was involved in regulating cell invasive ability. Transwell invasion assay showed that the invasion ability markedly increased in HCT116 and Caco-2 cells after silencing *FOXD3* compared with their control groups (Figure 3A and 3B). Epithelial-mesenchymal transition (EMT) is associated with cell invasion and tumor progression. Next, we examined the expression of EMT epithelial marker E-cadherin and mesenchymal marker N-cadherin in FOXD3 knockdown and control cells. Western blot results showed that *FOXD3* knockdown caused the marked decrease of E-cadherin but the obvious increase of N-cadherin in HCT116 and Caco-2 cells (Figure 3C and 3D). These results indicated that FOXD3 inhibited colon cancer cell invasion and the EMT process.

### FOXD3 inhibited EGFR/Ras/Raf/MEK/ERK signal pathway *in vitro*

**Figure 4:**
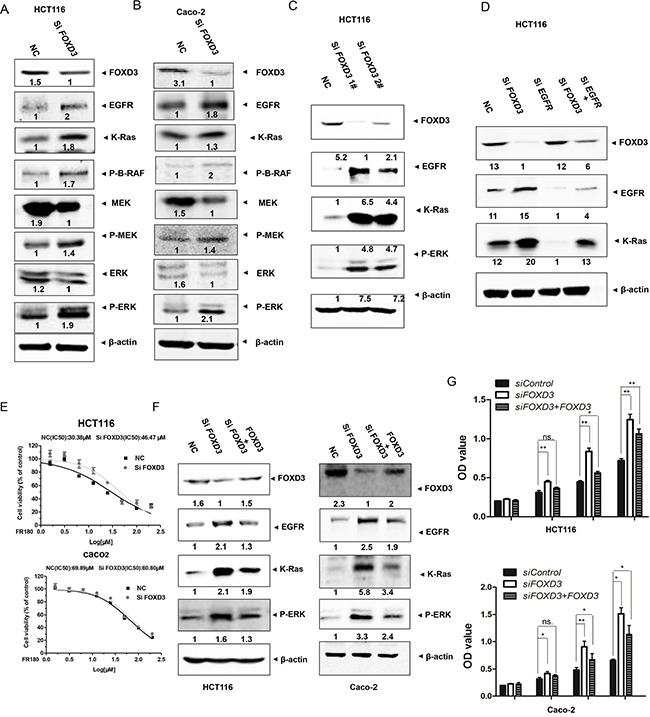
Knockdown FOXD3 activated EGFR/Ras/Raf/MEK/ERK signal pathway in the human colon cancer cells **A** and **B**. HCT116 and Caco-2 cells were treated with scrambled siRNA (control) or *FOXD3* siRNA for 72h and then collected for Western Blot analysis. Total protein was isolated from HCT116 and Caco-2 cells and analyzed by immunoblotting with the antibodies against EGFR, K-Ras, p-RAF, p-MEK, MEK, p-ERK and ERK. **C**. HCT116 cells were treated with scrambled siRNA (control) or *FOXD3* siRNA 1^#^ and 2^#^ for 72h and then collected for Western Blot analysis. **D**. HCT116 and Caco-2 cells were treated with *FOXD3* siRNA with or without EGFR siRNA for 72h. Total protein was isolated from cells and analyzed by immunoblotting with the antibodies against FOXD3, EGFR and K-Ras. **E**. Half maximal inhibitory concentration (IC50) of ERK inhibitor. Human colon cancer were transfected with scrambled siRNA (control) or *FOXD3* siRNA for 72h and then treated with various concentrations of ERK inhibitor for 24 h, and the IC50 values were determined by analyzing the data with GraphPad Prism 3.03. The X axes in each graph is presented as log10 values, and the data are plotted as the mean ± SD. *p<0.05, Si*FOXD3* versus control. **F**. Rescue blanket of blanket *FOXD3* siRNA-mediatedeffectsby reintroducing a FOXD3 expression vector in HCT116 and Caco-2 cells. Negative control cells and *FOXD3* knockdown cells transfected with or without blanket FOXD3 expression vector were collected for Western Blot analysis **G**. Cell viability of control cells and *FOXD3* knockdown cells transfected with or without FOXD3 expression vector was monitored by MTT assay in HCT116 and Caco-2 cells. Data are expressed as mean ± SD (n = 6).*p<0.05, **p<0.01.

The epidermal growth factor receptor (EGFR) and its downstream signaling pathways are involved in the development and progression of several human tumors, including colorectal cancer [14]. Moreover, FOXD3 expression was elevated following the targeted inhibition of the B-RAF-MEK (MAP/ERK kinase)-ERK (extracellular signal-regulated kinase)1/2 pathway in mutant B-RAF melanoma cells [15]. Therefore, we evaluated the effects of *FOXD3* knockdown on the activity of EGFR/Ras/Raf/MEK/ERK signal pathway. Western blot analyses showed that *FOXD3* knockdown increased the levels of EGFR and K-Ras in HCT116 and Caco-2 cells (Figure 4A and 4B). Moreover, the phosphorylation levels of B-Raf, MEK and ERK were increased in si*FOXD3* colon cancer cells compared to the normal cells (Figure 4A, 4B, 4C). To determine the effects of *FOXD3* knockdown on cell signaling pathways depending on EGFR, HCT116 cells were transfected with siRNA targeted against *EGFR*. Induction of K-ras by si*FOXD3* was blocked by EGFR inhibition (Figure 4D). Also, we used the selective inhibitor, PD98059, to inhibit MEK/ERK signaling-induced cell survival. The half maximal inhibitory concentration of inhibitor was markedly higher in *FOXD3* knockdown HCT116 colon cancer cells than the normal cells (Figure 4E). These results suggested that EGFR/Ras/Raf/MEK/ERK was downstream targets of the FOXD3 pathway in colon cancer cells.

In order to further confirm that FOXD3 was a suppressor of colon cancer cell proliferation, we performed rescue experiments by transfection of FOXD3 protein into *FOXD3* knockdown cells. Although FOXD3 rescue did not fully reverse the effects of its knockdown, the activated EGFR/Ras/Raf/MEK/ERK signal pathway in *FOXD3* knockdown cells was markedly inhibited (Figure 4F). Meanwhile, increased cell proliferation induced by *FOXD3* knockdown was also markedly reversed after restoring FOXD3 expression (Figure 4G).

### Knockdown *FOXD3* activated EGFR/Ras/Raf/MEK/ERK signal pathway in xenograft and human samples

**Figure 5:**
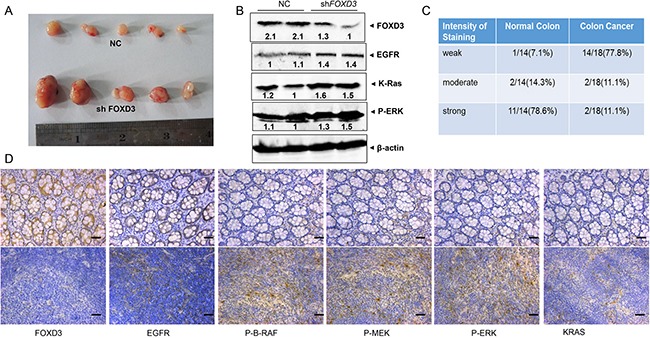
FOXD3 inhibited EGFR/Ras/Raf/MEK/ERK signal pathway in xenograft tumor and human samples **A**. Effects of FOXD3 knockdown on growth of HCT116 subcutaneous xenografts in vivo. Nude mice were injected with 10^6^ cells and tumors were removed at two weeks post injection; each experimental group contained 5 tumors. **B**. Total protein was isolated from mouse tumors and analyzed by immunoblotting with the antibodies against EGFR, K-Ras and p-ERK. **C**. The semi-quantitative analysis of FOXD3 immunohisochemical staining in human normal colon and cancer tissues. **D**. Immunohistochemical analysis of FOXD3, EGFR, K-Ras, p-BRAF, p-MEK, and p-ERK expression in human normal (upper) and colon cancer (lower) tissues. Typical fields of view are presented (Scale bar, 50μm; original magnification,×20).

To further investigate the effects of FOXD3 signaling on colon carcinogenesis *in vivo*, *FOXD3*-knockdown HCT116 stable cells were injected into the flanks of nude mice subcutaneously. As expected, *FOXD3*-knockdown HCT116 cells showed more tumorigenicity compared with the control groups. Meanwhile, our results showed that tumor size was clearly increased compared with those of the controls (Figure 5A). Western blot analysis from those tumor samples verified that FOXD3 protein expression was reduced in the knockdown tumor samples compared with the controls (Figure 5B). We also observed that the levels of EGFR, K-Ras and p-ERK1/2 were markedly increased in mouse tumor injected with *FOXD3*-knockdown HCT116 cells compared with the controls (Figure 5B).

In human colon cancer tissues, immuno histochemistry staining was used to measure the level of FOXD3 expression. As expected, FOXD3 expression was significantly reduced in the tumor tissues compared with the normal tissues (Figure 5C). 77.8% colon cancer tissues showed negative staining for FOXD3, while 78.6% normal tissues showed obvious positive staining (Figure 5D). Consistent with the previous results, tumor tissues showed more positive staining of EGFR, K-Ras, p-B-Raf, p-MEK and p-ERK1/2 compared with the normal colon tissues (Figure 5D).

## DISCUSSION

FOXD3 is an important stem cell factor expressed in many types of embryonic cells. A role for FOXD3 in development has been established. Also, FOXD3 was reported to participate in the carcinogenesis of several cancers as a potential tumor suppressor [9, 11]. In this study, we showed that FOXD3 worked as a suppressor for colon cancer formation. *FOXD3* knockdown dramatically increased human colon cancer cell proliferation i*n vitro and in vivo*, enhanced cell invasive ability and inhibited cell apoptosis. Moreover, we demonstrated that silencing *FOXD3* in human colon cancer cells markedly activated EGFR/Ras/Raf/MEK/ERK signal pathway. In human cancer tissue, FOXD3 expression was reduced while EGFR/Ras/Raf/MEK/ERK signal pathway was activated. Thus, we concluded that FOXD3 could suppress human colon formation by regulating EGFR/Ras/Raf/MEK/ERK signal pathway.

Colorectal cancer, like most solid tumors, results from uncontrolled growth of a small subpopulation of undifferentiated cancer stem cells. In this study, our results showed that *FOXD3* knockdown markedly promoted colon cancer cell proliferation and xenograft tumor formation. The previous studies demonstrated that FOXD3 overexpression significantly inhibits cell growth in lung cancer cells and induces a potent G0/G1 arrest in melanoma cells [16], which is consistent with our results. In addition, *FOXD3* deficiency was demonstrated to promote breast cancer progression by induction of epithelial-mesenchymal transition [17]. In contrast, FOXD3 overexpression inhibits the migration, invasion, and spheroid outgrowth of mutant B-RAF melanoma cells [18]. Similarly, in colon cancer, we found that *FOXD3* knockdown promoted cell migration and induced mesenchymal attributes. Thus, it is plausible that FOXD3 is a potential tumor suppressor in human colon cancer progression and represents a promising clinical prognostic marker and therapeutic target for this disease.

Hyperactivation of the Ras/Raf/MEK/ERK pathway is a driving force in many tumor types. BRAF mutations occur in 5~22% of all colorectal cancers. K-Ras mutation status is a strong predictive marker of resistance to EGFR-targeted therapy in patients with metastatic colorectal cancer. Although the EGFR-Ras-Raf-MEK-ERK signaling network has been identified as a novel target-based approach for cancer treatment [19], it is still unclear if the signal pathway is related with FOXD3. A previous study showed that FOXD3 expression is elevated following the targeted inhibition of the B-RAF-MEK (MAP/ERK kinase)-ERK (extracellular signal-regulated kinase) 1/2 pathway in mutant B-RAF melanoma cells. In our study, we demonstrated that *FOXD3* knockdown markedly activated EGFR-Ras-Raf-MEK-ERK signaling pathway in human colon cancer *in vitro and in vivo*. These data indicate that EGFR-Ras-Raf-MEK-ERK signaling pathway may be one of the important effectors of FOXD3 downstreams. However, it is still unclear if inhibition of FOXD3 by itself was able to induce colon tumorigenesis. The conditional knockout mice will be a useful model to discuss the role of FOXD3 in colon cancer. It is known that Wnt/β-cantenin signal pathway is closely associated with human colon cancer formation. Further investigation is needed to reveal the relations between FOXD3 and Wnt signal pathway.

In conclusion, *FOXD3* knockdown results in colon cancer cell proliferation and tumor formation, increased cell invasive ability and decreased cell apoptosis. Meanwhile, FOXD3 knockdown activated EGFR-Ras-Raf-MEK-ERK signaling pathway in human colon cancer cells and caner tissues. We believed that FOXD3 is likely to be an essential factor in protecting against human colon cancer formation, whose effects are mediated by EGFR-Ras-Raf-MEK-ERK signaling pathway.

## MATERIALS AND METHODS

### Cell lines

HCT116 and Caco-2 cells were purchased from ATCC. The stable cell lines were generated by integration of retroviral shRNA vectors specific for *FOXD3* or a control gene from OriGene (Rockville, MD). All cell lines were cultured in DMEM supplemented with 10% fetal bovine serum (Gibco).

### RNA interference and Realtime-PCR

Si*FOXD3* (sense strand, 1# 5′-GCAAUAG GGACGCGCCAAU-3′ 2# 5′-AGACGGCGCUCAUG AUGCA-3) was purchased from GenePharma Co., Ltd (Shanghai, China). FOXD3 SMART pool was purchased from Dharmacon/Thermo Fisher Scientific. Transfection of the siRNA oligonucleotide duplexes was performed in a 6-well plate (1 × 10^5^ cells per well) with Lipofectamine 2000 (Invitrogen, Inc.), using the methods recommended by the manufacturer. Using western blotting and real-time PCR, knockdown of *FOXD3* with siRNA was examined 72 h after siRNA transfection. PCR primer sets were designed using Primer Premier 5, and the sequences were as follows: *FOXD3*, 5-AGCAAGCCCAAGAATAGC-3 (forward) and 5-TCCAGGGTCCAGTAGTTG-3 (reverse). For rescue experiments, we transfected the FOXD3 plasmid to the cells after transfection of the siRNA oligonucleotide for 48hrs. Next, we collected the cells after 24 hours.

### MTT

Cells were cultured in 24-well plates with 0.5 ml medium per well at 37°C in a CO_2_ incubator. After transient transfection of the cells with *FOXD3* siRNA or control siRNA for 24h, 48h and 72h, the cells were incubated for an additional 2h after the addition of 50 μL/well of 10 mg/mL 3-(4,5-dimethyl-thiazol-2-yl)-2,5-diphenyltetrazolium bromide (MTT) at 37°C. The supernatant was aspirated, and the MTT-formazan crystals formed by metabolically viable cells were dissolved in 500 μl of DMSO. The absorbance was measured by a microplate reader at a wavelength of 570 nm.

### Cell cycle analysis

Cells were washed twice with cold PBS, resuspended in cold 70% ethanol and stored at 4°C overnight. For flow cytometry, cells were washed three times in 3 ml of cold PBS, then stained with a PI solution (50 μg/ml PI, 50 Units/ml Rnase) for at least 20 min at room temperature. Each assay was repeated for three times. Fold change values were represented as mean of the three experiments.

### Crystal violet staining

Cells seeded in triplicate into 96 well dishes with density of 5×10^4^ cells per well were cultured in a 37°C. Individual colonies were fixed and stained with a solution containing 0.2% crystal violet in 10% ethanol for 30 minutes. The amount of dye taken up by the monolayer is quantitated in a spectrophotometer or plate reader at 570 nm.

### Apoptosis assay

Cell apoptosis was detected by Annexin V-FITC/PI Apoptosis Detection Kit (Abcam, Cambridge, MA, USA) according to the manufacturer's protocols. The cells were washed in ice-cold PBS, resuspended in 200 μL of binding buffer and incubated with 5 μL Annexin V-FITC solution with 5 μl of propidium iodide (PI) for 15 min at 4°C in the dark. The results were analyzed by flow cytometry.

### Western blot analysis

Total protein from each sample was loaded and immune-blots were analyzed using primary antibodies specific for FOXD3, EGFR, K-RAS, p-B-RAF, p-MEK, MEK, ERK and p-ERK1/2 overnight at 4°C. After incubation with fluorescent labeled secondary antibody, specific signals for proteins were visualized by LI-COR Odyssey Infrared Imaging System.

### Xenograft experiments

Female BALB/c nude mice at the age of 5 weeks were prepared. The cells were implanted into nude mice at 2×10^6^ cells in 100μl per spot. Two weeks after injection, mice bearing tumors were sacrificed for Western Blot analysis.

### Immunohistochemical examination

Samples were fixed with 4% paraformaldehyde for 3 days and were then dehydrated through a graded series of ethanol, embedded in paraffin and sectioned at 4μm. These sections were deparaffinized by xylene, followed by rehydration through a graded series of ethanol, and then stained with rabbit polyclonal EGFR, K-RAS, p-B-RAF, p-MEK and p-ERK1/2 antibody (Santa Cruz, CA, USA). Sections were subsequently incubated for 1h with biotinylated goat anti-rabbit antibody IgG and then for 30m with Streptavidin-HRP peroxidase. Color reaction product was visualized by using diaminobenzidine (DAB)-H_2_O_2_ as substrate for peroxidase. All sections were counterstained with hematoxylin.

### Data collection and statistical analysis

The statistical data was obtained by GraphPad Prism 5.0 software. The results were expressed as the mean ± s.d. Statistical analysis was performed using two tailed, paired Student's t test. A p value of less than 0.05 was considered statistically significant.
